# Spiral growth of multicomponent crystals: theoretical aspects

**DOI:** 10.3389/fchem.2023.1189729

**Published:** 2023-05-12

**Authors:** Alexey Redkov

**Affiliations:** Laboratory of Structural and Phase Transitions, Institute for Problems in Mechanical Engineering RAS, Saint-Petersburg, Russia

**Keywords:** multicomponent, crystal, growth, theory, BCF, Chernov, Gilmer-Ghez-Cabrera, mechanism

## Abstract

This paper presents recent advances in the theory of multicomponent crystal growth from gas or solution, focusing on the most common step-flow mechanisms: Burton-Cabrera-Frank, Chernov, and Gilmer-Ghez-Cabrera. Analytical expressions for the spiral crystal growth rate are presented, taking into account the properties of all species involved in the growth process. The paper also outlines theoretical approaches to consider these mechanisms in multicomponent systems, providing a foundation for future developments and exploration of previously unexplored effects. Some special cases are discussed, including the formation of nanoislands of pure components on the surface and their self-organization, the impact of applied mechanical stress on the growth rate, and the mechanisms of its influence on growth kinetics. The growth due to chemical reactions on the surface is also considered. Possible future directions for developing the theory are outlined. A brief overview of numerical approaches and software codes that are useful in theoretical studies of crystal growth is also given.

## 1 Introduction

**FIGURE 1:**
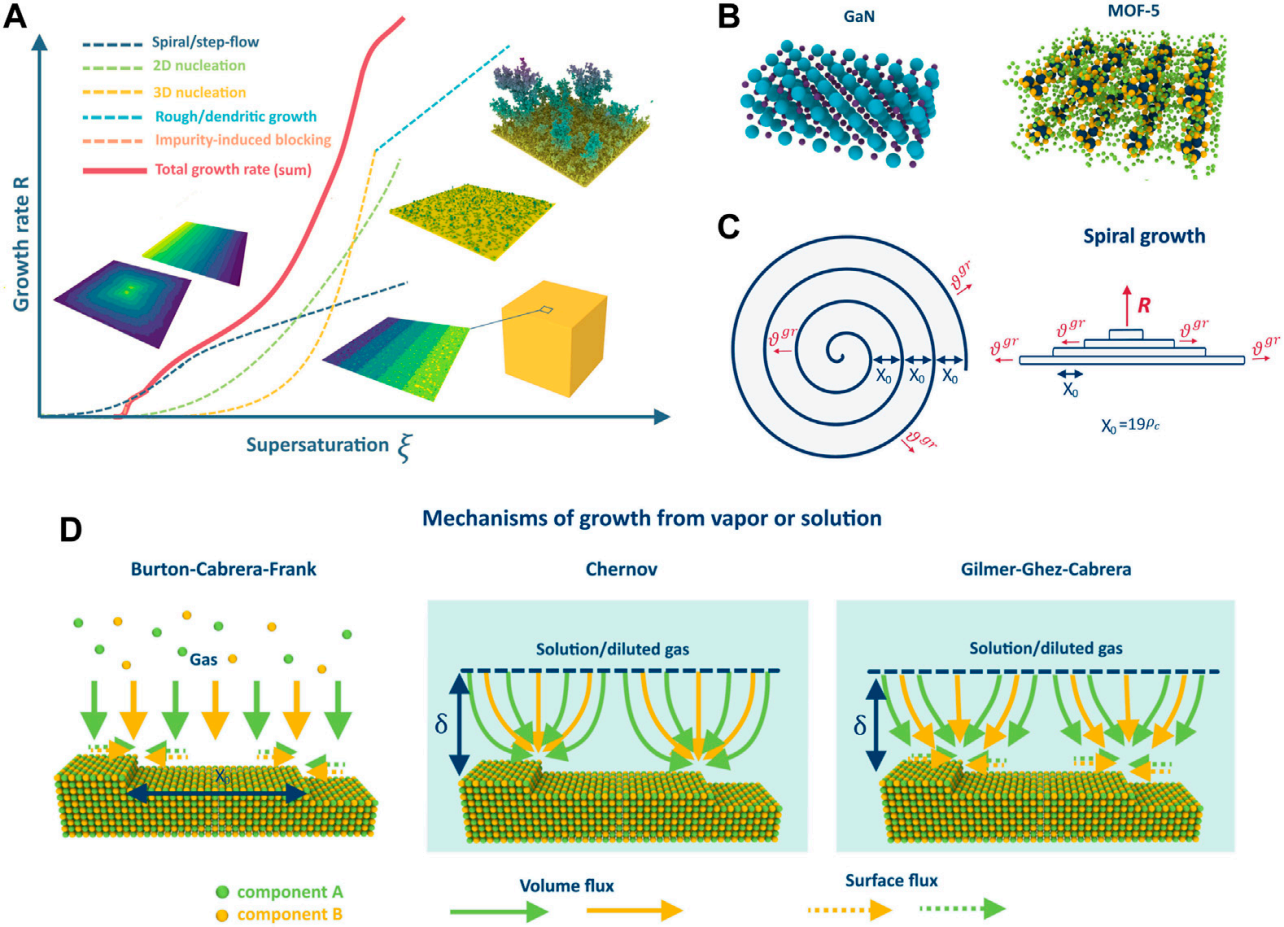
Dependence of the crystal growth rate on supersaturation in different growth regimes **(A)**; example of GaN and MOF-5 crystalline structures **(B)**; schematic representation of the spiral growth **(C)**; difference between the growth mechanisms on the example of 2-component AB crystal: BCF, Chernov and Gilmer-Ghez-Cabrera **(D)**.

In the past century, the field of crystal growth has emerged as a distinct and important branch of materials science, driven by the increasing demand for crystals in various technological applications such as electronics, optics, semiconductors, and more. While silicon formed the basis of modern electronics in the 20th century, high-tech industries are gradually transitioning towards more complex crystalline materials that can facilitate the creation of devices with superior characteristics. These materials include widebandgap semiconductors such as gallium and aluminum nitrides (GaN, AlN), which are widely used in optoelectronics (Kour et al., 2019), perovskite solar cells (Ansari et al., 2018; Jagadamma and Wang, 2021), prospective superconductors (Troyan et al., 2022), thermoelectric materials (Tohidi et al., 2022), and other functional compounds (Ramirez Reina et al., 2013; Wang et al., 2022), most of which consist of multiple components. A rapidly growing area of research is the development of metal-organic and other types of frameworks (Freund et al., 2021; Altaf et al., 2022) for gas capture, separation, storage (Wang et al., 2017), catalysis (Wang and Astruc, 2019), and various other applications. These materials are crystalline-like in structure and their growth mechanisms are similar to those of crystals, albeit more complex (See Figure 1B). They exhibit the same growth phenomena and in certain cases can be described by the same models and equations.

Note that there are various regimes of crystal growth, such as step-flow/spiral, nucleation, dendritic, and rough growth (refer to Figure 1A), and the realization of each depends on crystal properties and growth conditions. The growth regime determines the growth rate, crystal purity, defect concentration, and homogeneity, and a deep understanding of all the processes that occur during growth in different regimes is crucial for controllable synthesis of crystals with desired properties. This necessity has led to the rapid development of crystal growth theory and the detailed study of the growth regimes, especially step-flow/spiral and nucleation, since they are mostly used in the industry. The classical work by Burton, Cabrera, and Frank (BCF) (Burton et al., 1951) is a cornerstone of the theory that explains step-flow growth from vapors at low supersaturations due to screw dislocations. It revolutionized the understanding of crystal growth and allowed for the quantification of theoretical models and experiments. Since then, the theory has progressed, and various phenomena and effects have been investigated, such as the influence of elastic stresses (Van der Hoek et al., 1982), advacancies (Pimpinelli and Villain, 1994; Kosolobov, 2019; Rost et al., 2019), and impurities (Sangwal, 1996), among others. Analytical descriptions of different growth regimes and instabilities (Politi et al., 2000) have been proposed, including the formation of dendrites (Alexandrov and Galenko, 2021), the impact of island formation on terraces (Myers-Beaghton and Vvedensky, 1991), various types of self-organization, and other factors. There are numerous reviews and books which summarize the results (Bennema, 1984; Chernov, 2004; Uwaha, 2016) and different aspects of the theory, evolution of steps (Jeong and Williams, 1999) and kinks (Vekilov, 2007) on the surface. The BCF model has stimulated the development of other important growth models such as the Chernov model for growth from solutions or gas phase in the presence of a carrier gas (Chernov, 1961) and the mixed theory of Gilmer-Ghez-Cabrera (Gilmer et al., 1971). Together with nucleation theory (Dubrovskii, 2014), these models have facilitated the quantitative description of most gas or solution growth processes used in industry and accelerated the development of technologies and methods for obtaining low-defect crystalline materials like silicon. However, the majority of theories were developed using the classical model of the Kossel crystal. While it works well in single-component cases, it cannot always describe the growth of multicomponent crystals due to the different properties of the atoms (diffusion coefficients, atomic volumes, etc.). This calls for the extension of existing growth models and theory to describe crystal growth in multicomponent systems, which is in high demand in modern technology.

This perspective aims to discuss our recent developments in the theory of crystal growth in multicomponent systems via step-flow/spiral mechanisms, specifically focusing on the BCF (Burton et al., 1951), Chernov (Chernov 1961), and Gilmer-Ghez-Cabrera (Gilmer et al., 1971) models. We note that the multicomponent growth via the second important mechanism - nucleation, both classical and non-classical, is summarized elsewhere (Karthika et al., 2016; Kukushkin and Osipov, 1998). The article is organized as follows: In Section 2, a description of the mechanisms and their applicability limits is provided to help readers better understand which model is best suited for their experiments. Section 3 covers various aspects and effects that arise during growth via the step-flow mechanism. In Section 4, a discussion of growth mechanisms and effects is presented. The final expressions for the crystal growth rates are summarized in Supplementary Table S1. For the convenience of the readers, classical single-component expressions are also included. Additionally, the article provides a rigorous mathematical formulation of the models and a brief course on their solution in the Supplementary Materials, allowing readers to modify the models for their specific purposes if necessary. It should be noted that examples of the application of the proposed models to describe the growth of specific materials are provided in our referenced papers.

## 2 Crystal growth mechanisms

The problem of multicomponent spiral growth was formulated in (Redkov and Kukushkin, 2020) based on the classical BCF model. In this formulation, the crystal growth process involves the incorporation of building units into kinks at the spiral steps (see Figure 1C) on the surface in accordance with the following reaction:

$$\nu_{1}A_{1\left(g,s\right)}+\nu_{2}A_{2\left(g,s\right)}+\ldots +\nu_{N}A_{N\left(g,s\right)}↔C$$ (1)

where $A_{i\left(g,s\right)}$ represents the pure components (or building units), C is the solid crystalline phase; $\nu_{i}$ is the stoichiometric coefficient. The subscripts g) and s) denote the gas phase and solution, respectively, and N is the number of components. As previously mentioned, the mass transfer of components from the media to the kinks on the steps can occur through various mechanisms (as illustrated in Figure 1D), which will be discussed in detail later.

### 2.1 Burton-Cabrera-Frank mechanism

The Burton-Cabrera-Frank (BCF) model is commonly used to describe step-flow and spiral crystal growth from vapor. In the multicomponent version of this model (Redkov and Kukushkin, 2020), all components $A_{i\left(g\right)}$ that constitute the crystal are delivered to the terraces via uniform gas fluxes over the entire crystal surface. These components adhere to the surface and become adatoms (or admolecules), which then diffuse over the terraces towards existing steps and incorporate into them, thereby ensuring step motion and crystal growth. Adatoms can also evaporate back to the gas phase. Each type of adatom on the surface has unique properties, such as the surface diffusion coefficient and lifetime before evaporation. The strict mathematical formulation of this and the following models is presented in the Supplementary Materials. Here, we do not focus on mathematics, which is presented in detail in (Redkov and Kukushkin, 2020), and instead provide only the resulting analytical expression for the growth rate R (Supplementary Table S1). It turns out that the process of growth of a multicomponent crystal may be described by the same equation as a single-component crystal, using the so-called generalized coefficients of diffusion $D^{g}$ and incorporation $\beta^{g}$ , and generalized supersaturation $\xi^{g}$ . These coefficients are determined by the properties of the individual components and their surface concentration ratios. It can be seen from the definition of the generalized coefficients that the growth process is typically limited by the component that has the smallest product of diffusion or incorporation coefficient and concentration. It should be noted that this model assumes that: 1) reaction 1) occurs only and directly at the kinks, and not in the gas phase or on the terrace, which is valid for many growth processes; 2) the surface coverage is low, and the adatoms do not affect the diffusion or deposition of other atoms; 3) the step is always fully covered with kinks and is therefore a good sink for adatoms; note that the mechanisms of incorporation into kinks are presented in detail in the review (Vekilov PG. 2007); and 4) the rate of advancement of the step is sufficiently slow to neglect the convective flux of the adatoms towards the step.

### 2.2 Chernov mechanism

The Chernov model (Chernov, 1961) is commonly used to describe crystal growth from solution, but it is also applicable for growth from intrinsic vapors highly diluted by the carrier gas. Compared with the BCF model, the Chernov model has two key differences. First, the mass transfer in the volume is insufficient to maintain constant pressures/concentrations directly at the crystal surface, resulting in a depleted diffusion layer of some thickness δ in the mother liquid (or gas) phase. Second, incorporation occurs directly from the volume into the steps, bypassing the intermediate state on the terrace; thus, there is no surface diffusion. In the multicomponent version of this model, the crystal still grows in accordance with Reaction 1). At one of the boundaries of the depleted diffusion layer, constant concentrations of the components are maintained due to mixing or stirring (see Figure 1D). The components then diffuse through this layer and are incorporated directly into the steps one after another, resulting in crystal growth. The assumptions behind this model are almost the same as those in the BCF model (see section 2.1). However, it is important to note that this model fully neglects surface diffusion and is therefore only valid for systems with slow surface kinetics (e.g., owing to high surface diffusion barriers, surface reconstruction, coverage of the surface by an arbitrary adsorbate layer interfering with the surface diffusion of components, or other reasons). Furthermore, the thickness of the boundary layer is typically of the order of 10^−3^ to 10^−5^ cm, which is much smaller than the typical size of the growing crystal. As a result, some macro-diffusional fields may arise during growth, which must be considered to correctly describe the crystal growth over the entire surface.

### 2.3 Gilmer-Ghez-Cabrera mechanism

The Gilmer-Ghez-Cabrera model (Gilmer et al., 1971) is a significant generalization of the Burton-Cabrera-Frank and Chernov models. While the BCF and Chernov models consider only one type of diffusion, the Gilmer-Ghez-Cabrera mechanism takes into account both surface diffusion and volume diffusion of substances to the steps in the presence of a solvent or carrier gas. This is particularly important for multicomponent systems, where the flux of some components to the steps may be limited by volume diffusion, whereas for others, it may be limited by surface diffusion. The BCF and Chernov models may yield incorrect results in such cases because they consider only one type of flux (refer to Figure 1D). Notably, in the Gilmer-Ghez-Cabrera mechanism, adatom incorporation occurs through an intermediate adsorbed state on the terrace because there is no direct incorporation, as in the Chernov mechanism. It is worth mentioning that the Van Der Eerden model (Van Der Eerden, 1982) is the most complex model that accounts for both direct and indirect incorporation. However, this model has not yet been extended to multicomponent systems.

## 3 Different effects inherent to multicomponent systems

### 3.1 Nucleation of pure components on the crystal surface

**FIGURE 2:**
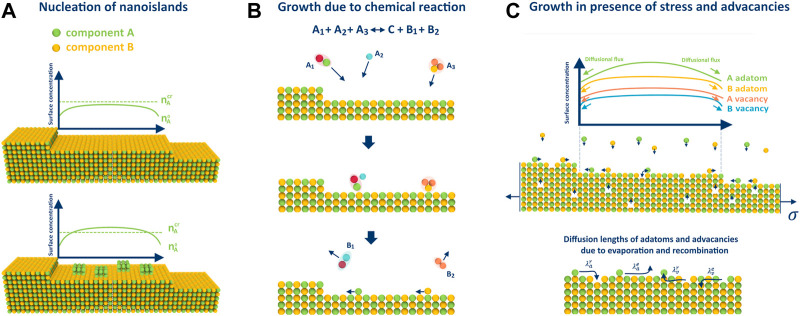
Illustrations for considered peculiarities of growth on the example of 2 component AB crystal: **(A)** nucleation of pure phase islands when one pressure exceeds the critical value, **(B)** adatom arrival on the surface through a chemical reaction, and **(C)** growth in the presence of advacancies, whose concentration is stress-dependent. Additionally, the figure displays different diffusion lengths of adatoms and advacancies due to recombination and evaporation.

In (Redkov and Kukushkin, 2020), the authors demonstrated that nucleation of islands consisting of pure components on the terraces between the steps is possible during step-flow growth, similar to the process of dew formation (refer to Figure 2A). In a subsequent study (Redkov and Kukushkin, 2021), it was shown that the precipitation of such islands can significantly impact the growth process, resulting in the formation of undesirable defects and inclusions in the crystal, and even cause different types of morphological instability (Bales and Zangwill, 1990). This phenomenon may occur when the surface concentration $n_{ai}\left(x\right)$ of a particular component on the surface between the steps exceeds the equilibrium single-component concentration $n_{ai}^{0}\left(x\right)$ required for nucleation. The authors also developed a dynamical criterion for determining whether nucleation of the *i*th component will occur during the BCF growth mode in a train of advancing steps.

$$\frac{\tau_{nucleation}^{i}}{\tau_{step}^{i}}<1$$ (2)

where $\tau_{nucleation}^{i}$ represents the time required for the formation of a single island at the surface concentration maintained at the terrace between subsequent steps, and $\tau_{step}^{i}$ , is the time required for the multicomponent step to traverse the terrace width and “sweep” it from adatoms, allowing for the nucleation process to restart.

### 3.2 Impact of chemical reactions

The growth of crystals in the presence of chemical reactions is common in modern synthesis techniques. For example, the metal-organic chemical vapor deposition (MOCVD) process used for GaN crystal growth typically involves surface chemistry (Tan et al., 2022). In (Redkov and Kukushkin, 2020), the authors investigated BCF-type growth in the most general case:

$$\nu_{1}A_{1\left(g,s\right)}+\nu_{2}A_{2\left(g,s\right)}+\ldots +\nu_{N}A_{N\left(g,s\right)}↔C+\nu_{N+1}B_{1\left(g,s\right)}+\ldots +\nu_{N+M}B_{M\left(g,s\right)}$$ (3)

when both the initial reagents and reaction products $A_{i\left(g,s\right)}$ , $B_{i\left(g,s\right)}$ are present and do not correspond to the building units of the crystal (see Figure 2B). It was shown, that in this complex case, the reaction must be broken down into sub-reactions, each bringing the building units/adatoms of the crystal to the surface. The growth in this case can still be described by the same single-component formula (see Supplementary Table S1), but if the thermal desorption of adatoms is slow and can be neglected in comparison to the fast reaction rate, the mean free paths and lifetimes of adatoms on the surface are primarily determined by reverse chemical sub-reactions, that is, by the pressures of the reaction products. Simultaneously, supersaturation will depend on the chemical reaction affinity, $\xi^{g}=\frac{K}{K_{eq}}-1$ , where $K_{eq}$ is the equilibrium constant of the reaction, and $K=\prod_{1}^{N}P_{Ai}^{\nu_{i}}/\prod_{1}^{M}P_{Bi}^{\nu_{N+i}}$ . This provides additional control levers for the crystal growth process.

### 3.3 Impact of mechanical stress and advacancies

Previous studies have established that mechanical stress may have a significant effect on crystal growth (Cabrera and Levine, 1956). In (Redkov and Kukushkin, 2022), it was demonstrated from a thermodynamic point of view that stress affects the growth of multicomponent crystals in the same way by reducing the effective supersaturation according to: $\xi^{g}\left(\sigma \right)=\xi^{g}\left(0\right)\left(1-\frac{\sigma \omega }{k_{B}T}\right)$ , where σ is the isotropic elastic stress and ω is the volume of the crystalline cell. This reduction factor can be crucial for the epitaxial growth of semiconductor or MOF-on-MOF heterostructures. Another important factor is the emergence and diffusion of vacancies on the crystal surface, which can recombine with adatoms and significantly affect the crystal growth or evaporation (Hirth, 1965; Pimpinelli and Villain, 1994; Sitnikov et al., 2017; Kosolobov, 2019). In (Redkov et al., 2020b; Redkov and Kukushkin, 2022), the authors extended the BCF model to the multicomponent case and accounted for the formation of additional vacancies resulting from applied stress (see Figure 2C). In this case the characteristic diffusion length, which determines the growth rate, is limited by the lowest diffusion lengths of adatoms and advacancies due to evaporation and recombination (see Figure 2C). It was demonstrated that the processes of adatom deposition, diffusion, and incorporation into the steps due to supersaturation in the gas phase are similar to the same processes for advacancies but due to applied tensile loads.

### 3.4 Morphological instability in multicomponent systems

The phenomenon of morphological instability was first introduced by Mullins and Sekerka (Mullins and Sekerka, 1963). It typically results in a change in the shape of a growing crystal or thin film, leading to irregular dendritic shapes or other inhomogeneities at a specific spatial wavelengths. In (Redkov et al., 2015; Kukushkin et al., 2014) the authors expanded the Mullins-Sekerka morphological stability theory to the growth of multicomponent strained thin films and spherical multicomponent particles. Their research showed that once a certain level of supersaturation is exceeded, the aforementioned shapes become unstable, leading to the emergence of irregular dendritic or undulating shapes. Analytical criteria were developed for this phenomenon, which connect the properties of different components (e.g., diffusion coefficients and atomic volumes), ratio of concentrations, and total supersaturation. This allows the selection of stable growth conditions for the crystal surface.

## 4 Discussion

The models presented here allow for the description of a wide range of growth processes from gases or solutions. They also provide a means for the experimental determination of all the kinetic coefficients for each component in complex multicomponent systems. As demonstrated in (Redkov and Kukushkin, 2020), the reaction 1) between adatoms at the kinks places a constraint only on the product of equilibrium concentrations $n_{ai}^{0}$ which should equal to the equilibrium constant $K_{eq}$ ; $\prod_{1}^{N}n_{ai}^{0}=K_{eq}$ , and on the stoichiometry of the fluxes of the components from terrace to kink and back, but it does not determine $n_{ai}^{0}$ themselves. This is in contrast to single-component systems, in which the equilibrium concentration is fixed at a constant temperature. Therefore, there are many possible pressure (or concentration) equilibrium sets at which a multicomponent system can exist. By selecting a set in which the pressure of one component is much lower than that of the others, one can ensure that this component limits the generalized coefficients $D^{g}$ and $\beta^{g}$ , which describe the dependence of the growth rate on supersaturation (see Supplementary Table S1). This means that by measuring this dependence at a series of different equilibrium pressure sets, it is possible to determine the individual kinetic coefficients of each component and use them to describe the growth at any other combination of pressures.

The criterion 3) presented in Sec. 3.1 shows that to eliminate the undesirable nucleation of pure components, the crystal should be grown at a pressure set where the surface concentrations of adatoms of each component do not exceed its critical value. This phenomenon can also be used for the controlled formation of self-organized quantum dots or nanowire growth in certain systems (Redkov and Kukushkin, 2021). In Sec. 3.2, BCF-growth when different types of adatoms are delivered to the surface by chemical reactions is considered. It differs from simple growth from vapor because chemical reactions alter the surface diffusion lengths of each component in comparison to thermal desorption, providing additional means to control the growth process. During a chemical reaction, the partial pressures of the reagents can be changed independently, allowing for individual control of supersaturation (chemical affinity), the mean free path of adatoms of each type, and their equilibrium concentrations. This enables control of the impact of each component on the generalized coefficients to ensure the maximum growth rate or to avoid undesirable phenomena, such as nucleation or instabilities. In Sec. 3.3, the impact of mechanical stress on the growth is considered. It is worth noting that mechanical stress are often present during the epitaxial growth of semiconductor thin films and heterostructures. The stress not only affect thermodynamics but also impact the kinetics of growth by determining the concentration of vacancies on the surface.

## 5 Future work

Although the mechanisms considered above are capable of describing numerous growth processes, significant gaps in knowledge and issues must be resolved to fully understand the multicomponent growth process. Further theoretical investigations could focus on several areas, including the impact of surface coverage of different components on mass transport (diffusion) both on the surface and in solution; the use of different adsorption isotherms for components to modify the relationship between surface supersaturation and pressure/concentration; the variation of Schwoebel barriers for each component, potentially affecting the distribution of adatoms; the detailed study of adatom incorporation into kinks, including the statistics of multicomponent kinks; the effect of surface reconstruction on diffusion mechanisms, which may depend on component concentration and growth rate; various morphological instabilities in multicomponent systems; the influence of impurities on incorporation or mass transfer of different components; anisotropic phenomena; the impact of convective flow of components during growth at higher supersaturations, when the step advancement rate cannot be ignored; and many other effects and their combinations.

## 6 Computer modeling

We note that in recent years, significant advancements have been made in high-performance modeling methods for multicomponent crystal structures and their growth. Some of these techniques include *ab initio* quantum mechanical (QM) modeling and stable structure prediction (Glass et al., 2006), molecular dynamics (MD) simulations (Salvalaglio et al., 2015; Joswiak et al., 2018), kinetic Monte Carlo (kMC) simulations (Andersen et al., 2019), phase-field modeling (PF) (Pierre-Louis, 2003; Gomez et al., 2019), and the level-set approach (Ratsch et al., 2002; Gibou and FedkiwOsher, 2018), among others. Each of these methods has its own temporal and spatial scales, strengths, and weaknesses (Miller, 2015), but all can be used to deepen our understanding of the growth process in multicomponent systems. For example, the quantum mechanical approach can be used to find the stable crystalline structure with a given composition and its surface reconstruction under specific growth conditions. See, e.g., the USPEX code (Glass et al., 2006; Kvashnin et al., 2019). The latter may significantly affect the diffusion of the different components on the surface. The QM approach is also commonly used to find *in silico* the activation energy of surface diffusion and desorption of atoms of various components (Won et al., 2009), which is needed to use the formulas provided in the previous sections. QM can also be used to analyze the formation energies of kinks of different types (Kuvadia and Doherty, 2011). Note that the concentration of kinks mainly determines the incorporation coefficients used in the theoretical models. The next scale is molecular dynamics, which may be used to analyze the statistics of kinks, steps, nucleation (Anwar and Zahn, 2011; Sosso et al., 2016), and the rates of incorporation of different components into the crystalline cell. We note that such phenomena can now be measured using experimental techniques (Dong et al., 2020). MD is also often used for the prediction of diffusion coefficients of components in solution (Celebi et al., 2021), which may be useful in the case of growth from solution by the Gilmer-Ghez-Cabrera and Chernov mechanisms. Note that for an accurate description of the processes using the MD method, machine learning potentials are often used, which are trained on quantum modeling data, even for crystals containing several components. See, for example, the MLIP code (Novikov et al., 2020). The Monte-Carlo method is capable of describing larger time and spatial scales, up to micrometers and milliseconds, respectively, even on personal computers. Precise modeling requires knowledge of all the probabilities of atomistic events occurring on a surface, which can be obtained through quantum mechanics or molecular dynamics. Recently, the authors of the CrystalGrower package (Anderson et al., 2017) implemented a unified kinetic three-dimensional partition model that can model the crystal habit and surface topology of any crystalline structure or MOF in different growth regimes, including nucleation and spiral growth. Note that various method combinations have also been developed, including a combination of Monte Carlo simulations with cellular automaton (Załuska-Kotur et al., 2021), which, while less accurate, can significantly speed up the performance and detect various macro-phenomena on the surface, such as different types of step instabilities and growth regimes. The phase-field approach is also noteworthy, as it allows for the effective study of macrosurface kinetics and spiral growth (Miura, 2015; Miura and Kobayashi, 2015; Nie et al., 2018). To provide a complete *in silico* description of macroscopic crystal growth or dissolution, a multiscale approach is often used, involving all the aforementioned techniques (Vvedensky, 2004; Miller, 2015) from nano-to microscale, simultaneously coupled with finite-element models. In (Elts et al., 2017), this approach was applied to the growth of multicomponent organic crystals. Furthermore, machine learning methods are being actively developed and hold promise for various applications, such as the search for stable crystalline structures (Kang et al., 2022), determination of kinetic parameters of adatoms on the surface (Martynec et al., 2021), and optimization of growth chambers (Schimmel et al., 2022). These new digital tools provide extensive information for the theoretical understanding of growth in multicomponent systems while also offering opportunities to discover new collective phenomena and effects and study them. They can also be successfully applied to solve the problems mentioned above.

## 7 Conclusion

In conclusion, we have summarized several growth mechanisms and effects involved in the growth of multicomponent crystals from vapors or solutions. Our findings demonstrate that growth can be described by the same equations as in the single-component case, with the use of generalized diffusion and incorporation coefficients based on the properties of each component. The results presented herein can be applied to a wide range of complex multicomponent systems, allowing the prediction of the growth rate based on growth conditions or, *vice versa*, the determination of individual kinetic properties of different components based on the dependence of the growth rate on conditions. We have also discussed the effects and peculiarities inherent to multicomponent systems, including the precipitation of nanoislands, effect of stresses, chemical reactions, and morphological instabilities. These phenomena can have a significant impact on the growth of multicomponent crystals or MOFs, making it crucial to understand their roles and the opportunities they provide for controlling the growth process in such complex systems. We presented approaches for accounting for new effects in theoretical equations, which enable the modification and extension of the theory to include additional factors. Lastly, we discussed some of the available numerical approaches and software packages, which make it possible to deepen knowledge and simplify the theoretical consideration of the crystal growth process.

## Data Availability

The original contributions presented in the study are included in the article/Supplementary Material, further inquiries can be directed to the corresponding author.
