# Supplementary material for: Spiral growth of multicomponent crystals: theoretical aspects
Source: Front Chem. 2023 May 12;11:1189729. doi: 10.3389/fchem.2023.1189729 (PMC10213516; doi:10.3389/fchem.2023.1189729)
Supplement: Supplementary file 1 [file DataSheet1.docx]

Supplementary Material

Spiral Growth of Multicomponent Crystals: Theoretical Aspects

Alexey Redkov *,

*** Correspondence:** [avredkov@gmail.com](mailto:avredkov@gmail.com)

**Table 1. Expressions for the spiral growth rate in different cases.**

| ***Notation*** | | |
| --- | --- | --- |
| *Related to the i-th component*  $p_{i}$ – actual pressure in gas phase  $p_{i0}$ – equilibrium pressure  $С_{i\infty}$ – concentration in the bulk gas/solution  $С_{i0}$ – equilibrium concentration  $n_{ai}^{0}$ – equilibrium surface concentration of adatoms  $n_{vi}^{0}$ – equilibrium surface concentration of advacancies  $D_{ai}$ – adatom surface diffusion coefficient  $D_{vi}$ – advacancy surface diffusion coefficient  $D_{Si}$ – volume diffusion coefficient (in solution)  $\tau_{ai}$ – lifetime of adatom on the surface due to evaporation (thermal desorption)  $\tau_{vi}$ – lifetime of advacancy on the surface due to evaporation to bulk crystal  $\lambda_{ai}^{e}=\sqrt{D_{ai}\tau_{ai}} , \lambda_{ai}^{r}$adatom diffusion length due to evaporation and recombination with advacancy respectively  $\lambda_{vi}^{r}$, $\lambda_{vi}^{e}$ advacancy diffusion length due recombination and evaporation, respectively  $\beta_{i}$ – adatom incorporation coefficient  $\omega_{i}$ – atomic volume  $\nu_{i}$ – stoichiometric coefficient  $\xi_{i}=\frac{p_{i}}{p_{i0}}-1$, $\xi_{i}=\frac{С_{i\infty}}{С_{i0}}-1$ supersaturation of the i-th component for the case of gas or solution, respectively | | $\Lambda_{i}$ *–* characteristic length for solution-adsorbate transition  $\Lambda_{si}$ *–* characteristic length for adsorbate-step transition (see Gilmer et al., 1971)  $k_{di}$*,*$k_{ri}$ *–* reaction rates of direct and reverse chemical reactions, which supplies/removes i-th component to the surface  $I_{i}$ – adatom-advacancy interchange parameter, see (Redkov and Kukushkin, 2022)  *General*  $N$ *–* number of initial reagents  M *–* number of reaction products  L – number of distinct crystal components (in case of chemical reaction)  $K=\prod_{i=1}^{N+M} ({p_{i})}^{{}_{i}}$  $K_{eq}$ *–* equilibrium constant  $\xi^{g}$ – multicomponent supersaturation  $\sigma$ – applied mechanical stress  $\delta$ – thickness of the boundary layer  $\omega=\sum_{i=1}^{L} \nu_{i}\omega_{i}$ – volume of crystalline cell  $\rho_{c}=\frac{\gamma\omega}{kT\xi^{g}}$ – critical radius of 2D-nucleus  $\gamma$ – surface energy of crystal  $x_{0}$ – interstep distance  $a$ *–* lattice parameter |
| ***Growth process*** | ***Expression for the spiral growth rate R and parameters for different cases*** | |
| ***BCF (vapor)*** | $\boldsymbol{R=}\frac{\boldsymbol{2}\boldsymbol{\omega}}{\boldsymbol{19}\boldsymbol{\rho}_{\boldsymbol{c}}}\frac{\boldsymbol{D}^{\boldsymbol{g}}\boldsymbol{\beta}^{\boldsymbol{g}}}{\boldsymbol{D}^{\boldsymbol{g}}\boldsymbol{+}\boldsymbol{\beta}^{\boldsymbol{g}}}\boldsymbol{\xi}^{\boldsymbol{g}}$ | |
| *Single-component**  *(Burton et al., 1951)* | $\xi^{g}=\frac{P}{{P\text{ }}_{0}}-1 D^{g}=\frac{\lambda_{a}}{D_{a}n_{a}^{0}\mathrm{th}\left( \frac{19\rho_{c}}{{2\lambda}} \right)} \beta^{g}=\beta\omega n_{a}^{0}$ $\lambda_{a}^{2}=D_{a}\tau_{a}$  (index *i* is omitted) | |
| *Single-component with chemical reaction*  *(Redkov and Kukushkin, 2020)* | $\xi^{g}=\frac{K}{K_{eq}}-1 \lambda_{a}^{2}=\frac{D_{a}}{k_{r}\prod_{1}^{M} P_{B_{i}}^{{}_{N+i}}}$  (index *i* is omitted) | |
| *Multicomponent*  *(Redkov and Kukushkin, 2020)* | $D^{g}=\left( \sum_{i=1}^{L} \frac{{\nu_{i}}^{2}\lambda_{i}}{\left. D_{ai}n_{ai}^{0}th(\frac{19\rho_{c}}{{2\lambda}_{i}} \right)} \right)^{-1}$ $\beta^{g}=\left( \sum_{i=1}^{L} \frac{{\nu_{i}}^{2}}{\beta_{i}\omega_{i}n_{ai}^{0}} \right)^{-1}$  $\xi^{g}=\sum_{i=1}^{N} {{}_{i}\xi}_{i}$ $\lambda_{i}^{2}=D_{ai}\tau_{i}$ | |
| *Multicomponent with chemical reaction*  *(Redkov and Kukushkin, 2020)* | $D^{g}=\left( \sum_{i=1}^{L} \frac{{\nu_{i}}^{2}\lambda_{i}}{\left. D_{ai}n_{ai}^{0}th(\frac{19\rho_{c}}{{2\lambda}_{i}} \right)} \right)^{-1}$ $\beta^{g}=\left( \sum_{i=1}^{L} \frac{{\nu_{i}}^{2}}{\beta_{i}\omega_{i}n_{ai}^{0}} \right)^{-1}$  $\xi^{g}=\frac{K}{K_{eq}}-1$ $\lambda_{i}^{2}=\frac{D_{i}}{k_{ri}\prod_{m=1}^{M_{i}} P_{B_{m}}}$  $M_{i}$ is the number of products of the subreaction, which provides i-th component, $B_{m}$ is its products, see details in (Redkov and Kukushkin, 2020) | |
| **BCF in presence of stress and advacancies (vapor)** | $\boldsymbol{R=}\frac{\mathbf{2}\boldsymbol{\omega}}{\mathbf{19}\boldsymbol{\rho}_{\boldsymbol{c}}}\left( {\boldsymbol{D}_{\boldsymbol{a}}}^{\boldsymbol{g}}\left( \boldsymbol{\xi}^{\boldsymbol{g}}\mathbf{-}\frac{\boldsymbol{\sigma}\boldsymbol{\omega}}{\boldsymbol{k}_{\boldsymbol{B}}\boldsymbol{T}}\mathbf{-}\boldsymbol{I}_{\boldsymbol{a}} \right)\mathbf{-}{\boldsymbol{D}_{\boldsymbol{v}}}^{\boldsymbol{g}}\left( \frac{\boldsymbol{\sigma}\boldsymbol{\omega}}{\boldsymbol{k}_{\boldsymbol{B}}\boldsymbol{T}}\mathbf{-}\boldsymbol{I}_{\boldsymbol{v}} \right) \right)$ | |
| Multicompontent  *(Redkov and Kukushkin, 2022)* | ${D_{a}}^{g}=\left( \sum_{i=1}^{N} \frac{\lambda_{i}{\nu_{i}}^{2}}{D_{ai}n_{ai}^{0}\mathrm{th}\left( \frac{19\rho_{c}}{2\lambda_{i}} \right)} \right)^{-1}$ ${D_{v}}^{g}=\left( \sum_{i=1}^{N} \frac{\lambda_{i}{\nu_{i}}^{2}}{D_{vi}n_{vi}^{0}\mathrm{th}\left( \frac{19\rho_{c}}{2\lambda_{i}} \right)} \right)^{-1}$  $I_{v}=\sum_{i=1}^{N} I_{i}\nu_{i}n_{ai}^{0}\tau_{vi} I_{a}=\sum_{i=1}^{N} I_{i}\nu_{i}n_{vi}^{0}\tau_{ai}$  $\lambda_{i}^{2}={(\frac{1}{{\lambda_{ai}^{e}}^{2}}+\frac{1}{{\lambda_{vi}^{e}}^{2}}+\frac{1}{{\lambda_{ai}^{r}}^{2}}+\frac{1}{{\lambda_{vi}^{r}}^{2}})}^{-1}$ $\xi^{g}=\sum_{i=1}^{N} {{}_{i}\xi}_{i}$ | |
| **Chernov***  **(gas/solution)** | $\boldsymbol{R=}\frac{\boldsymbol{a}\boldsymbol{\xi}^{\boldsymbol{g}}\boldsymbol{\omega}}{\boldsymbol{19}\boldsymbol{\rho}_{\boldsymbol{c}}}\left( \frac{\boldsymbol{\pi}\boldsymbol{\beta}^{\boldsymbol{g}}{\boldsymbol{D}^{\boldsymbol{g}}}}{\boldsymbol{D}^{\boldsymbol{g}}\boldsymbol{+}\boldsymbol{\beta}^{\boldsymbol{g}}\boldsymbol{a}\ln\left( \frac{\boldsymbol{19}\boldsymbol{\rho}_{\boldsymbol{c}}}{\boldsymbol{\pi a}}\boldsymbol{sh(}\frac{\boldsymbol{\pi}\boldsymbol{\delta}}{\boldsymbol{19}\boldsymbol{\rho}_{\boldsymbol{c}}}\boldsymbol{)} \right)} \right)$ | |
| Single-component  (Chernov, 1961) | $D^{g}=D_{S}С_{0}$ $\beta^{g}=\beta\omegaС_{0}$ $\xi^{g}=\frac{С_{\infty}}{С_{0}}-1$  (index *i* is omitted) | |
| Multicomponent | $D^{g}=\left( \sum_{i=1}^{N} \frac{{\nu_{i}}^{2}}{D_{Si}С_{i0}} \right)^{-1}$ $\beta^{g}=\left( \sum_{i=1}^{N} \frac{{\nu_{i}}^{2}}{\beta_{i}\omega_{i}С_{i0}} \right)^{-1}$ $\xi^{g}=\sum_{i=1}^{N} {{}_{i}\xi}_{i}$ | |
| **Gilmer-Ghez-Cabrera (gas/solution)** | $\boldsymbol{R=}\frac{\boldsymbol{D}^{\boldsymbol{g}}\boldsymbol{(19}\boldsymbol{\rho}_{\boldsymbol{c}}\boldsymbol{,}\boldsymbol{\delta}\boldsymbol{)} \boldsymbol{\xi}^{\boldsymbol{g}}\boldsymbol{\omega}}{\boldsymbol{19}\boldsymbol{\rho}_{\boldsymbol{c}}}$ | |
| Single-component  (Gilmer et al., 1971) | $D^{g}\left( l,\delta\right)=\frac{C_{0}D_{S}l}{\Lambda}\left[ 1+\frac{\delta}{\Lambda}+\frac{\Lambda_{s}l}{\lambda^{2}}+2\sum_{k=1}^{\infty} \frac{\alpha_{k}+\frac{\lambda}{\Lambda}\tanh\frac{\alpha_{k}\delta}{\lambda}}{\alpha_{k}\left( {\alpha_{k}}^{2}+\alpha_{k}\frac{\lambda}{\Lambda}\tanh\frac{\alpha_{k}\delta}{\lambda}+1 \right)} \right]^{-1}$  $\alpha_{k}=2\pi k\lambda/l$ $\xi^{g}=\frac{С_{\infty}}{С_{0}}-1$ (index *i* is omitted) | |
| Multicomponent | $D^{g}\left( l,\delta\right)=\left( \sum_{i=1}^{N} \frac{{\nu_{i}}^{2}\Lambda_{i}}{C_{i0}D_{Si}l\left[ 1+\frac{\delta}{\Lambda_{i}}+\frac{\Lambda_{si}l}{{\lambda_{i}}^{2}}+2\sum_{k=1}^{\infty} \frac{\alpha_{ik}+\frac{\lambda_{i}}{\Lambda_{i}}\tanh\frac{\alpha_{ik}\delta}{\lambda_{i}}}{\alpha_{ik}\left( {\alpha_{ik}}^{2}+\alpha_{ik}\frac{\lambda_{i}}{\Lambda_{i}}\tanh\frac{\alpha_{ik}\delta}{\lambda_{i}}+1 \right)} \right]^{-1}} \right)^{-1}$  $\alpha_{ik}=2\pi k\lambda_{i}/l$ $\xi^{g}=\sum_{i=1}^{N} {{}_{i}\xi}_{i}$ | |
| *Note that original models were modified by adding coefficients of incorporation $\beta$, by using a single notation, and by taking into account corrected value for the distance between steps $y_{0}=19\rho_{c}$. See (Redkov and Kukushkin, 2020; Cabrera and Levine, 1961) | | |

# Mathematical formulation of the models

## Burton-Cabrera-Frank model

The problem formulation is described in detail in (Redkov and Kukushkin, 2020) and can be summarized as follows. The multicomponent flat crystal surface consists of terraces separated by equidistant steps with a distance of $x_{0}$ between them, which are covered with kinks. There are constant partial pressures ($p_{i}$) of different components over the entire surface, resulting in uniform fluxes ($J_{i}$) of these components from the gas phase to the terraces. Adatoms diffuse over the surface towards the steps and can either incorporate into kinks or desorb. Adatoms of different components only interact with each other at kink positions, incorporating into them according to the stoichiometric ratio. Each component has its own kinetic parameters, including diffusion coefficients, lifetimes, and incorporation coefficients. The distribution of adatoms of each component over the terrace $n_{ai}\left( x \right)$ between the steps in a quasistationary approach can be described by a system of N one-dimensional diffusion equations (one for each component, see Fig. 1S):

$\left\{ \begin{aligned} D_{a1}\frac{\partial^{2}n_{a1}\left( x \right)}{{\partial x}^{2}}-\frac{n_{a1}\left( x \right)}{\tau_{a1}}+J_{a1}=0 \\ D_{a2}\frac{\partial^{2}n_{a2}\left( x \right)}{{\partial x}^{2}}-\frac{n_{a2}\left( x \right)}{\tau_{a2}}+J_{a2}=0 \\ \ldots\\ D_{aN}\frac{\partial^{2}n_{aN}\left( x \right)}{{\partial x}^{2}}-\frac{n_{aN}\left( x \right)}{\tau_{aN}}+J_{aN}=0 \end{aligned} \right.$ (s1)

The value of $J_{ai}$ can be determined using the principle of detailed balance, which is given by $J_{ai}=\frac{p_{i}}{p_{i0}}\frac{n_{ai}^{0}}{\tau_{ai}}$. We consider the following boundary conditions: at the center of the terrace the surface flux is zero due to symmetry, so $\frac{dn_{ai}\left( x \right)}{dx}=\left. 0 \right|_{x=0}$*;* at the step, the flux can be determined by either diffusion or incorporation into the step, so we have ${D_{ai} \frac{dn_{ai}\left( x \right)}{dx}\left. =\beta_{i}\omega_{i}(n_{ai}\left( x \right)-n_{ai}^{0}) \right|}_{x=\frac{x_{0}}{2}}$ (where the steps are located at $\pm\frac{x_{0}}{2}$ positions). Solving the system of one-dimensional diffusion equations for each component yields a solution for the distribution functions of adatoms over the surface, allowing us to find the gradients of concentration near the steps and therefore the diffusion fluxes towards the steps. It should be noted, that these fluxes are interdependent which is determined by the stoichiometricity of the crystal. A chain of transformations, as described in (Redkov and Kukushkin, 2020) should be taken into account. This yields the total flux of building units towards the steps and an analytical equation for the dependence of the rate of advancement of the group of steps $\vartheta^{gr}$, which involves the generalized values of diffusion and incorporation coefficients:

$${\vartheta^{gr}}= \frac{2\xi^{g}}{n_{C0}}\left( \frac{D^{g}\beta^{g}}{D^{g}+\beta^{g}} \right)$$

where $D^{g}=\left( \sum_{i=1}^{N} \frac{{\nu_{i}}^{2}\lambda_{ai}}{\left. D_{ai}n_{ai}^{0}th(\frac{19\rho_{c}}{{2\lambda}_{ai}} \right)} \right)^{-1}$ and $\beta^{g}=\left( \sum_{i=1}^{N} \frac{{\nu_{i}}^{2}}{\beta_{i}\omega_{i}n_{ai}^{0}} \right)^{-1}$, 1/$n_{C0}$ is the surface area occupied by the crystalline cell (see Burton et al. 1951). The total multicomponent supersaturation $\xi^{g}$ is also taken into account, which is proportional to the chemical affinity of reaction (1). The value of $\xi^{g}$ can be calculated using the expression:

$$\xi^{g}=\sum_{i=1}^{N} \nu_{i}\xi_{i}\approx\sum_{i=1}^{N} ln({(\frac{p_{i}}{p_{i0}})}^{{}_{i}})\approx\prod_{i=1}^{N} ({\frac{p_{i}}{p_{i0}})}^{{}_{i}}-1=\frac{K}{K_{eq}}-1$$

The total spiral growth rate of the crystal can be determined from the rate of advancement of a group of equidistant steps using the formula given by (Burton et al., 1951). The formula is:

$$R=\frac{{2\omega}}{19\rho_{c}}\left( \frac{D^{g}\beta^{g}}{D^{g}+\beta^{g}} \right)\xi^{g}$$

where $\rho_{c}$ represents the critical radius of a 2D-nucleus. The details and different peculiarities of such systems can be found in (Redkov and Kukushkin, 2020).


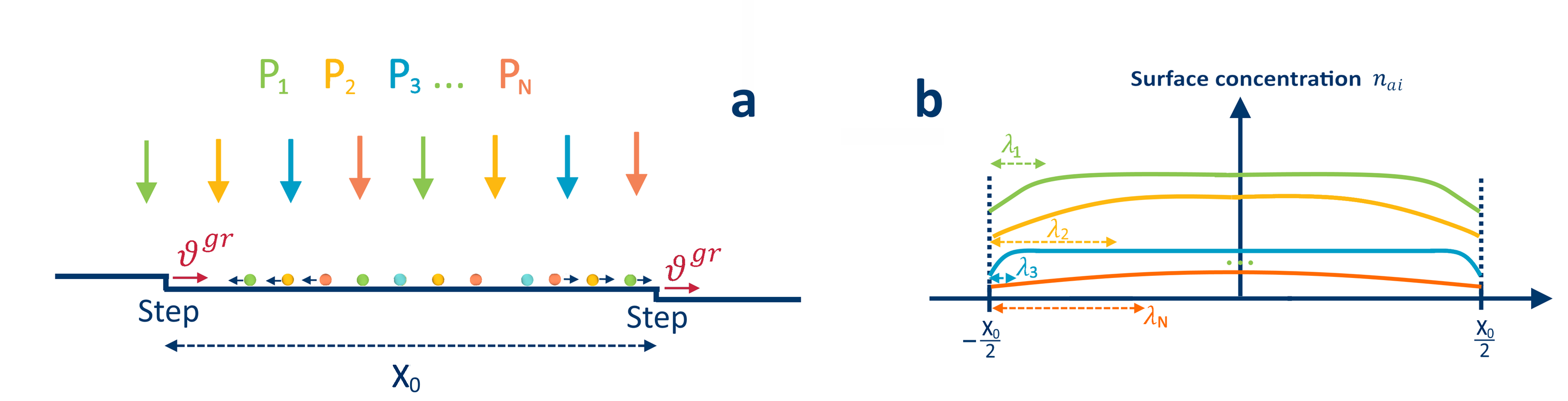


**Supplementary Figure 1.** Representation of the BCF mechanism. There are uniform fluxes of different components to the terraces with a group of equidistant steps. The diffusion of adatoms to the steps results in the advancement of the steps with a rate $\vartheta^{gr}$ (a); surface distribution functions $n_{ai}\left( x \right)$ of adatoms of different types, which are dependent on the fluxes, diffusion and evaporation rates (b).

## Chernov mechanism

The rate of multicomponent crystal growth in the Chernov mechanism (Chernov, 1961) can be determined by obtaining the distribution functions of the different components within the diffusional boundary layer and calculating the fluxes toward the steps. This is illustrated in Figure 2s. To facilitate calculations, a Cartesian coordinate system is employed, where the x-axis is aligned with the plane of the crystal surface, the y-axis is perpendicular to it, and the z-axis is aligned with one of the steps.


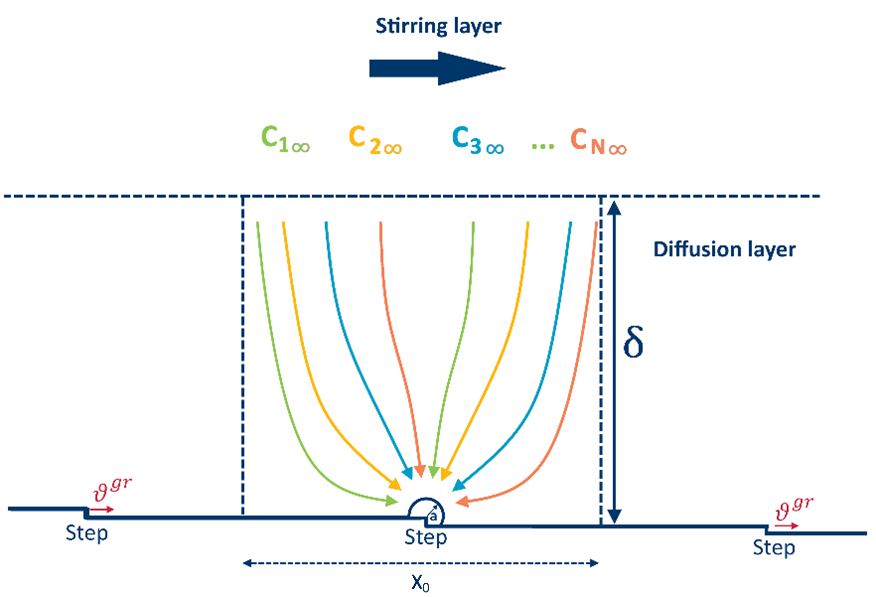


**Supplementary Figure 2.** The Chernov mechanism. The system has constant concentrations of different components, denoted as $С_{i\infty}$, in the stirring layer. A diffusional flux through the boundary layer of thickness δ is directed toward the steps on the terraces. This flux results in the advancement of the steps with a rate denoted as $\vartheta^{gr}$.

One may write a quasistationary system of N two-dimensional diffusion equations for the concentration of each component in the boundary diffusion layer over the surface $С_{i}(x,y)$:

$\left\{ \begin{aligned} \frac{d^{2}С_{1}}{d^{2}r}+\frac{1}{r}\frac{С_{1}}{\mathrm{dr}}=0 \\ \frac{d^{2}С_{2}}{d^{2}r}+\frac{1}{r}\frac{С_{2}}{\mathrm{dr}}=0 \\ \ldots\\ \frac{d^{2}С_{2}}{d^{2}r}+\frac{1}{r}\frac{С_{2}}{\mathrm{dr}}=0 \end{aligned} \right.$ (s2)

By solving this system around the step with a set of following boundary conditions: directly at the step $\left. D_{Si} \frac{d С_{i}}{dr}=\beta_{i}\omega_{i}(С_{i}-С_{i0}) \right|_{r=a/\pi}$ ($r=\sqrt{x^{2}+y^{2}}$), condition at the boundary of the diffusion layer $С_{i}\left( 0,\delta\right)=С_{i\infty}$ and the condition along a line connecting points equidistant from two steps which is $\frac{\partialС_{i}\left( {\mp x}_{0}/2,y \right)}{\partial x}=0$, distribution functions of each component can be obtained. Here, $С_{i0}$ is the equilibrium concentration of the i-th component, $С_{i\infty}$ is its actual concentration in the stirring layer, $D_{Si}$ are the volume diffusion coefficients, $a$ - is the lattice parameter. Knowing the distribution functions one can find the fluxes toward the steps. Then by applying the stoichiometric rule and a chain of transformations as was done in (Redkov and Kukushkin, 2020) one may find the final expression for the rate of advancement of the group of equidistant steps:

$$\vartheta^{gr}(x_{0},\delta)=\left( \frac{\pi\beta^{g}{D^{g}}}{D^{g}+\beta^{g}a\ln\left( \frac{x_{0}}{\pi a}sh(\frac{\pi\delta}{x_{0}}) \right)} \right)\xi^{g}\omega$$

where generalized diffusion and incorporation coefficients can be expressed as follows:

$D^{g}=\left( \sum_{i=1}^{N} \frac{{\nu_{i}}^{2}}{D_{Si}С_{i0}} \right)^{-1}$ $\beta^{g}=\left( \sum_{i=1}^{N} \frac{{\nu_{i}}^{2}}{\beta_{i}\omega_{i}С_{i0}} \right)^{-1}$

Now, knowing the rate of advancement of the group of parallel steps, it is easy to calculate the rate of spiral growth R.

$$R=\frac{a{\vartheta^{gr}(19\rho_{c},\delta)}}{19\rho_{c}}=\frac{a\xi^{g}\omega}{19\rho_{c}}\left( \frac{\pi\beta^{g}{D^{g}}}{D^{g}+\beta^{g}a\ln\left( \frac{19\rho_{c}}{\pi a}sh(\frac{\pi\delta}{19\rho_{c}}) \right)} \right)$$

Note that in the limit of a single-component system, this expression also coincides with the classical one (Chernov, 1961).

## Gilmer-Ghez-Cabrera mechanism

As before, we consider the growth of a multicomponent crystal according to reaction (1), where each component has its own kinetic parameters (volume and surface diffusion coefficients, surface lifetimes, concentrations in mother solution). In this process simultaneous diffusion of components in both the volume and surface occurs. It should be noted that in the Gilmer-Ghez-Cabrera mechanism adatom incorporation occurs through an intermediate adsorbed state on the terrace, there is no direct incorporation as in the Chernov mechanism. A schematic representation of the Gilmer-Ghez-Cabrera mechanism is illustrated in Figure 3s.


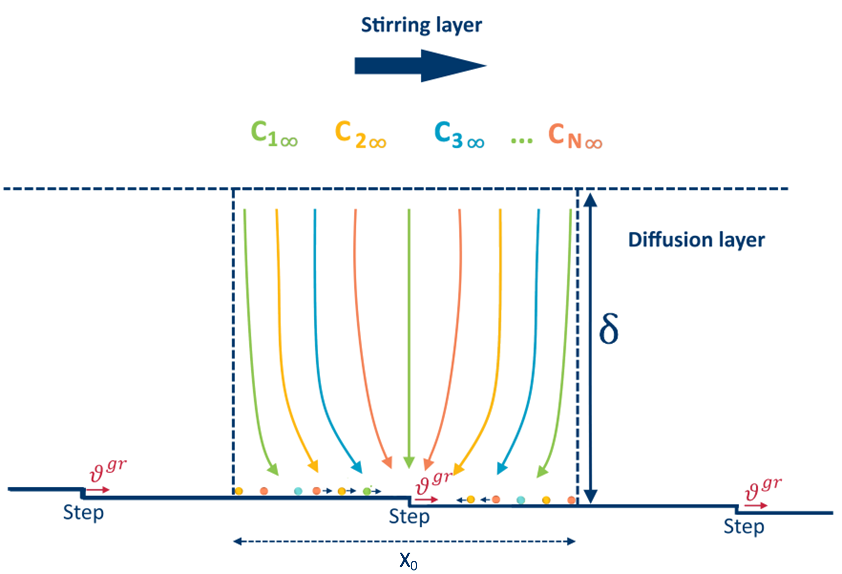


**Supplementary Figure 3.** The Gilmer-Ghez-Cabrera mechanism. As in Chernov mechanism, the system has constant concentrations of different components, denoted as $С_{i\infty}$, in the stirring layer. A diffusional flux flows through the boundary layer of thickness δ is directed to the terraces. Surface diffusion of deposited adatoms to the steps results in the advancement of the latter with a rate denoted as $\vartheta^{gr}$.

To find the crystal growth rate according to the mechanism under consideration, it is necessary to solve the following complex mathematical problem for the distribution function of each of the components both on the crystal surface and in the bulk diffusion layer above:

$\left\{ \begin{aligned} D_{ai}\frac{\partial^{2}n_{ai}(x)}{\partial x^{2}}+D_{Si}\left. \frac{\partialС_{i}}{\partial y} \right|_{y=0}=0, x\neq0 \\ \frac{\partial^{2}С_{i}(x,y)}{\partial x^{2}}+\frac{\partial^{2}С_{i}(x,y)}{\partial y^{2}}=0, y>0 \\ D_{Si}\left. \frac{\partialС_{i}(x,y)}{\partial y} \right|_{y=0}=\left. \frac{D_{Si}}{\Lambda_{i}}С_{i}(x,0) \right|_{y=0}-\frac{n_{ai}(x)}{\tau_{i}}, y=0 \end{aligned} \right.$ (s3)

where $\Lambda_{i}$ is the parameter describing the transition of the atom of the i-th component from the solution to the adsorbed state, which in certain cases can be comparable to the mean free path in the volume (Gilmer et al., 1971). The system takes into account the continuity equation directly on the surface. The boundary conditions for $С_{i}(x,y)$ at the boundary of the diffusion layer and along a line connecting points equidistant from two steps are the same as in the Chernov mechanism. The boundary condition for the surface diffusion flux of adatoms at the step is the following $j_{s}=D_{ai}/\Lambda_{si}(n_{ai}(0)$- $n_{ai}^{0})$, where $\Lambda_{si}$ is the parameter describing the “step”-“adsorbed state” transition (see Gilmer et al, 1971). By solving the system for each type of species involved, it is possible to determine $С_{i}(x,y)$, $n_{ai}$(x), and the total surface flux of the components towards the step. Then, by carrying out a chain of transformations similar to those in (Redkov, 2020), final expressions for the dependence of the growth rate on supersaturation can be found:

$$R=\frac{D^{g}(\rho_{c},\delta) \xi^{g}\omega}{19\rho_{c}}$$

where generalized diffusion coefficient $D^{g}$ can be expressed as:

$$D^{g}\left( l,\delta\right)=\left( \sum_{1}^{N} \frac{{\nu_{i}}^{2}\Lambda_{i}}{C_{i0}D_{Si}l\left[ 1+\frac{\delta}{\Lambda_{i}}+\frac{\Lambda_{si}l}{{\lambda_{i}}^{2}}+2\sum_{k=1}^{\infty} \frac{\alpha_{ik}+\frac{\lambda_{i}}{\Lambda_{i}}\tanh\frac{\alpha_{ik}\delta}{\lambda_{i}}}{\alpha_{ik}\left( {\alpha_{ik}}^{2}+\alpha_{ik}\frac{\lambda_{i}}{\Lambda_{i}}\tanh\frac{\alpha_{ik}\delta}{\lambda_{i}}+1 \right)} \right]^{-1}} \right)^{-1}$$

and coefficient $\alpha_{ik}$ inside square brackets equals to $2\pi k\lambda_{i}/l$. $l$ is the interstep distance. Note that in the limit of the single-component system this equation reduces to the one obtained in the (Gilmer et al., 1971).
